# MMP-9 affects dural cell composition and granulocyte accumulation during neuroinflammation

**DOI:** 10.3389/fimmu.2025.1713166

**Published:** 2025-12-11

**Authors:** Sheng-Hsiang Shen, Hanna Gerwien, Miriam Burmeister, Yi-Ling Tsang, I-Na Lu, Anna-Lena Börsch, Tushar Deshpande, Lydia Sorokin, Gerd Meyer zu Hörste

**Affiliations:** 1 Department of Neurology, Medical Faculty, University Hospital Muenster, Münster, Germany; 2 Institute of Physiological Chemistry and Pathobiochemistry, and Cells-in-Motion Interfaculty Centre (CiMIC), University of Muenster, Münster, Germany; 3 Department of Pediatric Hematology and Oncology, University Hospital Muenster, Münster, Germany

**Keywords:** matrix metalloproteinase-9 (MMP-9), neuroinflammation, meningeal immunity, dura mater, neutrophil granulocytes, experimental autoimmune encephalomyelitis (EAE), single-nucleus RNA sequencing (snRNA-seq)

## Abstract

**Introduction:**

Matrix metalloproteinases MMP-2 and MMP-9 modulate inflammatory processes at the blood–brain barrier (BBB), but their presence and function in the dura mater remain unclear. We therefore investigated their contribution to immune regulation at this site during neuroinflammation.

**Methods:**

We analyzed dura mater from naïve and experimental autoimmune encephalomyelitis (EAE) mice using immunofluorescence, gelatin zymography, flow cytometry, and single-nucleus RNA sequencing (snRNA-seq). Comparisons were performed between wild-type (WT) and *Mmp9*-/- mice to define gelatinase expression, cellular sources, transcriptional alterations, and immune cell dynamics.

**Results:**

Pro- and activated-MMP-2 and MMP-9 were detectable in naïve dura, with selective upregulation of MMP-9 during EAE. *Mmp9*-/- mice demonstrated delayed EAE onset but more severe disease at peak. SnRNA-seq revealed that *Mmp9* deficiency altered endothelial transcriptional programs, especially in venous endothelial cells, and promoted expansion of granulocyte populations with mature neutrophil signatures. Flow cytometry and immunofluorescence confirmed increased Ly6C⁺Ly6G⁺ neutrophils in *Mmp9*-/- dura during EAE.

**Discussion:**

Our findings indicate that MMP-9 regulates immune cell composition and activation within the dura during neuroinflammation. While *Mmp9* deficiency delays disease onset, it enhances neutrophil accumulation and inflammatory responses at this CNS border, suggesting a previously unrecognized, potentially anti-inflammatory role for MMP-9 in the dura.

## Introduction

The gelatinases, matrix-metalloproteinases (MMP)-2 and MMP-9, are expressed by immune and stromal cells (1–4) and were traditionally considered to function in extracellular matrix (ECM) remodeling during development and disease (5). However, increasing data suggest a major role *in vivo* in the fine tuning of inflammatory processes (6–8). We have previously demonstrated gelatinase expression by both immune cells and astrocytes during neuroinflammation (2, 9, 10). Astrocyte-derived gelatinases modulate cell-cell interactions and chemokine signaling at the blood-brain barrier (BBB) (8) particularly at the parenchymal/astroglial border of postcapillary venules, and are essential for T cell infiltration into the parenchyma of the central nervous system (CNS) during experimental autoimmune encephalomyelitis (EAE) (9). Whether MMP-2 and MMP-9 are also present and contribute to neuroinflammation in the dura layer of the meninges, which forms a border tissue of the CNS, remains unknown.

The multi-layered meninges have recently gained substantial interest due to the controversial discussion of their involvement in modulating CNS homeostasis and inflammation (11–13). The meninges ‘encase’ the brain parenchyma, forming an interface for neuroimmune interactions similar to that of BBB at the level of smaller vessels. The dura, the outermost layer of meninges, has been suggested to be an immune relevant tissue, containing not only lymphatic vessels but also dural-associated lymphoid tissues (14–16). Notably, in homeostatic conditions the dura contains a rich repertoire of immune cells, including B cell populations at different developmental stages, contributing to CNS immune surveillance and immune defense (17–19). Mechanisms controlling the composition of dura leukocytes remain poorly understood (20) and a potential contribution of gelatinases has not yet been studied.

Here, we demonstrate that both MMP-2 and MMP-9 are expressed and activated in the naïve WT murine dura, with specific upregulation of pro- and activated-MMP-9 during EAE. To identify potential gelatinase functions in the dura, single-nuclei transcriptomes were generated from dural cells of naïve and EAE wild-type C57BL/6 mice (WT), EAE and MMP-9 deficient (EAE *Mmp9*^-/-^) mice. We identified cell clusters annotated as dural fibroblasts and osteoblasts as the primary cellular sources of *Mmp2*, while *Mmp9* was predominantly expressed by granulocytes and osteoclasts. Focusing on *Mmp9* due to its selective upregulation in EAE, a dural venous endothelial subtype was identified in *Mmp9*^-/-^ dura which displayed the greatest transcriptional alterations associated with enhanced inflammatory responses during EAE. Furthermore, a granulocyte subtype with mature neutrophil features was observed at higher proportions in the dura of *Mmp9^-/-^* mice during EAE. The accumulation of the overall neutrophil population in these conditions was confirmed by flow cytometry and immunofluorescence staining. MMP-9, thus, may modulate immune responses within the dura during EAE, potentially by regulating the inflammatory milieu and myeloid cell populations.

## Materials and methods

### Animal subjects

Animal housing and ethical approval: All animal experiments were performed in accordance with institutional and national guidelines for animal welfare and approved by the Landesamt für Verbraucherschutz (LAVE) (Nordrhein-Westfalen, Deutschland; permit number AZ2024-42). Mice were housed under standard laboratory conditions (12 h light/dark cycle, temperature 20–24°C, relative humidity 45–65%) with free access to food and water. Animals were kept in groups in individually ventilated cages with appropriate bedding and environmental enrichment. All efforts were made to minimize animal suffering, and experiments were conducted in line with the 3Rs principle.

Euthanasia: No anesthesia was used in this study. For sacrifice, animals were euthanized by controlled exposure to carbon dioxide (CO_2_) in accordance with the American Veterinary Medical Association (AVMA) Guidelines for the Euthanasia of Animals (2020). CO_2_ was introduced into the chamber at a displacement rate of 30–70% of the chamber volume per minute. Cervical dislocation was not applied in order to preserve the brain tissue for subsequent analyses.

### Gelatin zymography

For gelatin zymography, whole dura preparations of PBS perfused mice or small pieces of tissue isolated from new-born mice (40 mg) were meshed using 2.8 mm ceramic beads (Precellys) in 200 µl lysis buffer (cOmpleteTM EDTA-free proteinase Inhibitor cocktail (Roche), 0.1% Triton X-100 in PBS). 120 µl were recovered for gelatin prepurification by incubation with gelatin- Sepharose-4B for 20 min on a rotating wheel (21). Total purified protein was taken up in sample buffer (2x) and run on a 10% polyacrylamide gel containing 1 mg/ml gelatin under non-reducing conditions. After the electrophoresis, the gels were washed with H_2_O containing 2.5% Triton X-100 (v/v) for 30 min followed by 2.5% Triton X-100 in H_2_O for another 30 min to renature the gelatinases. Subsequently, the gels were placed in developing buffer (TBS, 5 mM CaCl_2_, and 0.02% NP-40) overnight at 37°C. The next day, gels were fixed in acetic acid:ethanol:H_2_O (10:50:40) for 30 min at RT and washed for 30 min in acetic acid:methanol:H_2_O (10:50:40). Gels were then stained with Coomassie Brilliant Blue for 1h at RT, followed by washing of the gels acid:methanol:H_2_O (10:50:40) for 120 min or until cleaved gelatin bands became visible. Areas of cleaved gelatin showed as clear bands that correspond to the activated MMP subunits; recombinant mouse MMP-2 and MMP-9 were used as molecular weight controls.

### Immunofluorescence staining

Mice were euthanized, in case of EAE at the peak of disease severity; skull and attached dura were collected and fixed overnight in 2% paraformaldehyde (PFA) at 4°C. The next day samples were washed with PBS and the dura was manually peeled from the skull using a binocular.

For immunofluorescent staining whole mount dura were blocked for 2-3h in 1% BSA solution containing 1% Triton-X in a humidified chamber at room temperature. Subsequently, primary antibodies were applied overnight at 4°C. Primary antibodies included rat anti-mouse CD45.2 (30G12), Rabbit anti-mouse laminin 111 (22), rat anti-mouse CD4 (RM4-5) Alexa Fluor647 (BD Bioscience), Isolectin GS-B4 Alexa Fluor 647 (23), FITC rat anti-mouse Ly6G (24) and rat anti-mouse PECAM-1 clone: 2H8 (25). After washing thrice secondary antibodies including Alexa Fluor488 donkey anti-rat IgG (Invitrogen), Alexa Fluor594 donkey anti-rabbit IgG (Invitrogen) and Alexa Fluor647 goat anti-hamster IgG (Abcam) were applied together with DAPI (4′,6-diamidino-2-phenylindole) (1 μg/ml) to visualize nuclei. Sections were examined with LSM800 AxioVert microscope.

### Image processing and quantification

For vessel density quantification, whole-mount dura tile images were analyzed using Imaris 10.0.1. Images were first stitched using ZEN Blue software, then converted from.czi to.ims format using the Imaris File Converter. The resulting.ims files were opened in Imaris for 3D analysis. Surface rendering was applied to segment a region of interest (ROI). The PECAM-1 (CD31) channel was selected for analysis. Surface smoothing and background-subtracted thresholding were used to refine the segmentation. Surfaces were classified, and the total PECAM-1-positive surface area within the ROI was measured. This total area was then normalized to the size of the ROI. The normalized vessel density data were plotted and statistically analyzed using GraphPad Prism.

### Flow cytometric characterization of dural neutrophils in EAE dura

Dura mater was carefully peeled from naïve or EAE-induced C57BL/6 or *Mmp9^-/-^* mice and immediately transferred to ice-cold PBS. Tissue was enzymatically dissociated in Collagenase type I and DNase I (37°C, 45 min, gentle agitation), then mechanically triturated and passed through a 70 µm cell strainer to obtain single-cell suspensions. Cells were washed and resuspended in FACS buffer (PBS, 2% FCS). For surface staining, cells were incubated for 30 min at 4°C with fluorochrome-conjugated antibodies (CD45-PerCP (BD Bioscience), CD45-FITC (BD Bioscience), CD4-PE (BD Bioscience), CD8-BV605 (BD Bioscience), B220-BV421 (BD Bioscience), CD11b-FITC (BD Bioscience), CD11c-APC (eBioscience), Ly6C-PE (BioLegend), Ly6G-APC (BioLegend). After staining, cells were washed, filtered, and analyzed on a BD FACSCelesta flow cytometer. Neutrophils were quantified using a standard gating strategy (FSC/SSC to exclude debris, singlet discrimination, life/dead and leukocyte (CD45) gate, and Ly6G^4^/Ly6C^4^ subsets). All steps were performed on ice or at 4°C unless noted otherwise.

### Mice, EAE induction, and dura isolation for snRNA-seq

**Figure 1:**
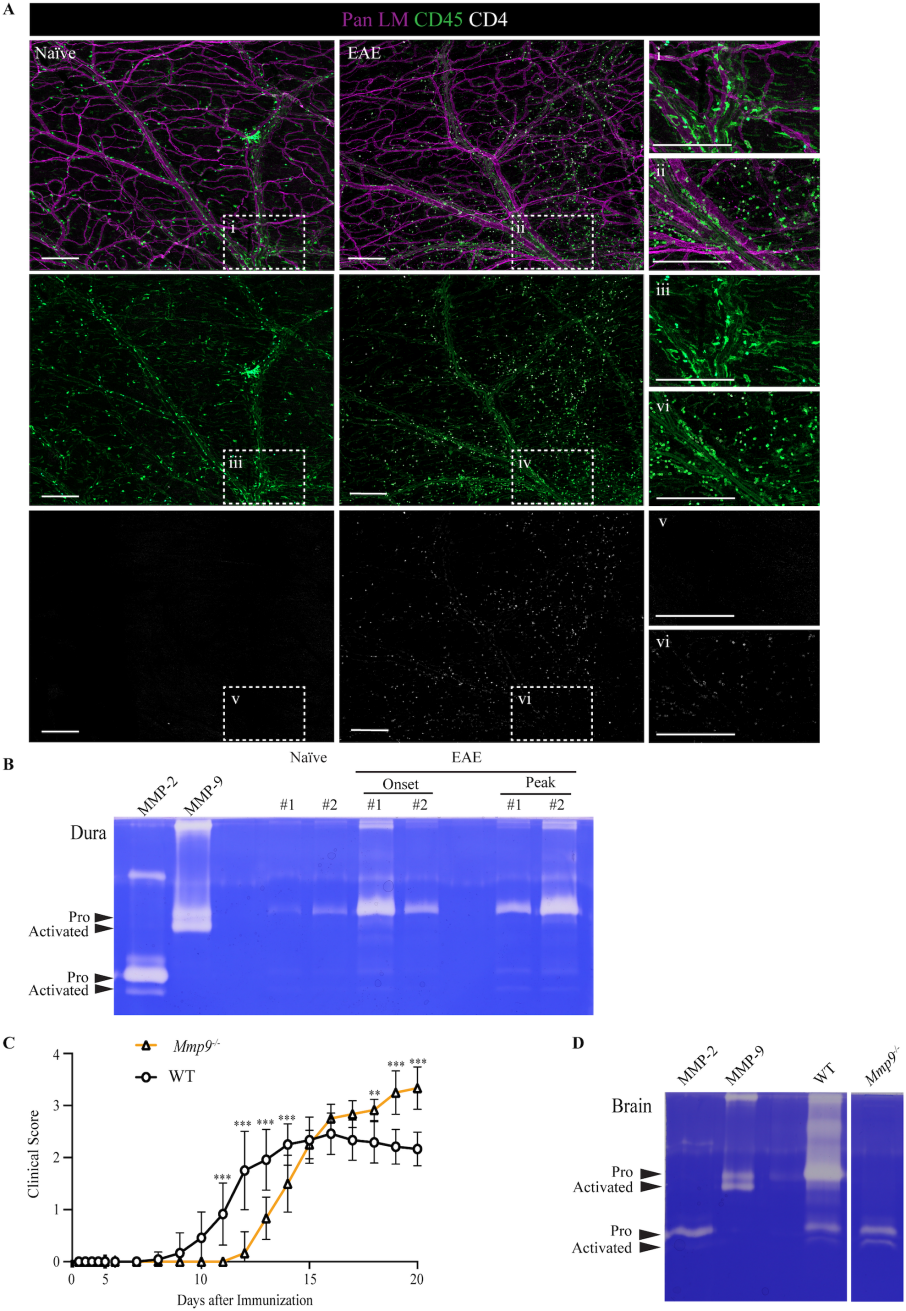
MMP-2 and MMP-9 are expressed and activated in the murine dura. **(A)** Whole-mount immunofluorescence of dura mater from naïve and peak EAE WT mice stained for pan laminin (Pan LM, basement membranes, magenta), CD45 (immune cells, green), and CD4 (CD4^+^ T cells, white). Scale bar = 250 µm. **(B)** Gelatin zymography of dura extracts from naïve, EAE onset, and peak EAE WT mice (two biological replicates per condition) showing pro- and activated forms of MMP-2 and MMP-9. **(C)** Clinical EAE scores in WT and *Mmp9^-/-^* mice; data shown are means ± SEM from 12 WT and 6 *Mmp9*^-/-^ mice and is one example of 3 separate experiments using a total of 24 WT and 23 *Mmp9*^-/-^ mice. Statistical analysis was performed using the Mann-Whitney U test, **p < 0.01; ***p < 0.001. **(D)** Gelatin zymography of brain extracts from EAE WT and EAE *Mmp9^-/-^* mice.

EAE was induced in C57BL/6 mice from Charles River Laboratories (Cologne, Germany) and MMP-9 (*Mmp9*^-/-^) deficient mice bred on a C57BL/6 background (8). Female mice aged 8–12 weeks were immunized with MOG peptide (MOG_35-55_, 112.5 µg per mouse, Rudolf Volkmer, Charite Berlin, Germany) and *Mycobacterium tuberculosis* H37RA extract (5 mg ml^-1^, BD) emulsified in complete Freund’s adjuvant (150 µl per mouse). On days 0 and 2, pertussis toxin (200 ng per mouse) was injected intravenously. Mice were monitored daily, and disease severity was assigned as stage 1 (flaccid tail), stage 2 (hindlimb weakness), stage 3 (severe hindlimb weakness), stage 4 (hind quarter paralysis), and stage 5 (forelimb weakness). EAE incidence in WT and *Mmp9^-/-^* mice was comparable. Only the mice that developed EAE were included in the experiment and in the statistical analyses. For snRNA-seq, the dura tissue was collected from naïve WT mice and from EAE samples at 14 days after immunization, when WT and *Mmp9^-/-^* mice had similar disease scores (**Figure 1C**). Under terminal anesthesia, the mice were intracardially perfused with PBS, and the dura was peeled off from the dissected skull caps using fine forceps under a binocular. Dura preparations were kept at 4°C in PBS before nuclei extraction for snRNA-seq.

### Nuclei preparation for snRNA-seq

Nuclei extraction was performed according to the Milteny Biotec nuclei extraction protocol (Catalog no. 130-128-024). Four freshly collected duras from each experimental condition were pooled. Hence, the single nuclei data are derived from multiple biological replicates that were pooled and processed together. Pooled samples were transferred to lysis buffer (RNA inhibitor (0.2 U/µl) in nuclei extraction buffer) and single nuclei suspensions were generated using a gentleMACS dissociator with gentleMACS program 4C_nuclei_1 at 4°C. To minimize the debris contamination, the resulting single nuclei preparations were passed through a 70 µm as well as 30 µm cell strainers and a resuspension buffer (PBS with 0.1% ultrapure BSA and 0.2 U/µl RNase inhibitor) was used between the filtration steps. Subsequently, individual single nuclei preparations were stained with trypan blue and manually inspected in a Fuchs-Rosenthal chamber for determining the total number of the intact nuclei. A total of 10,000 nuclei per condition were used for downstream procedures including single nuclei libraries construction and sequencing.

### Generation of single nuclei libraries and sequencing

Single nuclei preparations were loaded onto the Chromium Single Cell Controller utilizing the Chromium Single Cell 3’ Library & Gel Bead Kit with v3.1 chemistry (10X Genomics). Quantification was performed with precision using the TapeStation High Sensitivity D1000 kit (Agilent Technologies) to ascertain the average fragment size and library concentration. Subsequently, the libraries underwent normalization, dilution, and were sequenced on a local Nextseq 2000 using the P3–100 cycle kit with a 28-10-10–90 read setup. Processing of sequencing data was performed with the cellranger pipeline v7.0.1 (10X Genomics) in accordance with the manufacturer’s instructions. Briefly, raw bcl files were analyzed using the cellranger mkfastq pipeline and subsequent read alignments and transcript counting were done individually for each sample with standard parameters.

### Data analysis of single-nuclei RNA-sequencing

*CellBender* was employed for individual datasets to remove background noise (26) prior to downstream bioinformatic analysis using the R-package *Seurat* v4.4.1 (27) in datasets of EAE *Mmp9*^-/-^, EAE *Mmp2*^-/-^ and WT samples, as well as a naïve murine dura were integrated after SCTransform (28). Low-quality cells (min.cell = 3) and cell doublets (scDblFinder.class = singlet) were excluded by filtering cells with few genes (<2% percentile), high number of genes (>97% percentile), high mitochondrial percentages (>5%), high percentages of erythrocytes (>2%), and high ribosomal percentages (>5%). The datasets were merged and normalized using *SCTransform*. The total remaining cell number for further analysis was 24219. To integrate the datasets and correct for batch effects, cells were aligned between different conditions with *Harmony* (29). Clusters were identified by the “FindNeighbors”and “FindClusters” (with resolution of 0.5) functions in *Seurat* v. 4.4.1 and Uniform Manifold Approximation and Projection (UMAP) was performed with Harmony embeddings to visualize the results. “FindMarker” function (Wilcoxon Rank Sum test) in *Seurat* v. 4.4.1 was applied to determine marker genes for each cell cluster. Four cell clusters were excluded for downstream analyses: a cluster expressing *Tph1*, thus likely representing pinealocytes; a cluster expressing *Aqp4*, thus likely representing astrocytes; a cluster expressing *Ttr*, thus likely representing epithelial-like cells, and a cluster expressing mainly ribosomal and mitochondrial RNA, thus representing low quality cells. Re-clustering was performed for the subset dataset (“FindClusters”; with a resolution of 0.37). To visualize individual cell transcriptomes, Uniform Manifold Approximation and Projection (UMAP) was performed with Harmony embeddings. We manually inspected the top DE genes of each cluster in a literature search cell cluster annotation. To determine DE genes between EAE conditions, scaled data were split by condition (WT, *Mmp2*^-/-^ and *Mmp9*^-/-^) and the “FindMarker” function in *Seurat* v.4.4.1 (Wilcoxon Rank Sum test) was applied. DotPlots and FeaturePlots were created using the internal visualization functions of *Seurat* v.4.4.1. Volcano plots of DE genes were created using *ggplot2* v.3.5.1 (30), and for GO enrichment analysis, the package *ClusterProfiler* v.4.12.0 (31) was used. Pseudotime analysis was performed on the vascular endothelial cell clusters subset from the combined dataset using *monocle3* (32) with default settings.

## Results

### Pro- and activated MMP-2 and MMP-9 are expressed in the dura of naïve and EAE mice

We first assessed whether gelatinases are expressed and activated in the dura of the meninges. Dura samples were isolated from naïve and peak EAE WT mice and whole-mounted immunofluorescently stained with pan laminin (Pan LM) antibody to label all basement membranes, anti-CD45 to mark immune cells and anti-CD4 to identify CD4^+^ T cells (**Figure 1A**). In separate stainings, immunofluorescence staining for CD206 and F4/80 was performed to identify resident and total macrophages, respectively (**Supplementary Figure 1**). In naïve dura, the majority of CD45^+^ immune cells exhibited an elongated morphology typical of resident macrophages, consistent with the high number and morphology of CD206^+^ resident macrophages (**Supplementary Figures 1A, B**), and with virtually no CD4^+^ T cells present. By contrast, at peak EAE CD4^+^ T cells were detectable throughout the dura but remained low in number compared with total CD45^+^ (**Figure 1A**) and CD206^+^ cells (**Supplementary Figure 1C**). Gelatin zymography of dura extracts confirmed the expression of pro-forms of MMP-2 and MMP-9 even in non-diseased tissue (**Figure 1B**). During EAE, both pro- and activated forms of MMP-9, and to a lesser extent MMP-2, were more abundant (**Figure 1B**), as previously reported for EAE brain tissues (9, 33). Thus, while both MMP-2 and MMP-9 are active in the dura under homeostatic conditions, activated MMP-9 is preferentially increased in the presence of abundant immune cell infiltration during EAE (onset and peak). The expression and activation pattern of gelatinases in the inflamed dura, thus, is similar to that of postcapillary venules at the border to the brain parenchyma (8, 9, 33). Given the selective upregulation of pro- and activated-MMP-9 in EAE dura, we focused subsequent analyses on *Mmp9^-/-^* dura to investigate its cellular origin/s and potential role/s in meningeal neuroinflammation.

We first analyzed the EAE disease course in *Mmp9^-/-^* mice, revealing delayed onset of symptoms compared to WT controls (**Figure 1C**), implicating MMP-9 in early disease processes. However, these mice ultimately developed more severe disease at later stages. To assess potential compensatory mechanisms, we performed gelatin zymography on brain extracts from WT and *Mmp9^-/-^* adult mice (**Figure 1D**), confirming the absence of MMP-9 and no compensatory upregulation or activation of MMP-2 in the *Mmp9^-/-^* samples. These data indicate that the altered EAE kinetics in *Mmp9^-/-^* mice are not due to increased MMP-2 activity.

### SnRNA-seq reveals cellular heterogeneity of murine dura

We next tested which cells in the dura express the gelatinases at the transcriptional level. As MMP-9 was specifically upregulated in EAE, snRNA-seq was performed on all nuclei of freshly dissected dura from WT and *Mmp9^-/-^* mice at day 14 after EAE induction, when they exhibited similar disease scores (**Figure 1C**). Since *Mmp9^-^*^/-^ mice exhibited delayed onset of EAE symptoms compared to WT, but ultimately developed more severe disease, analysis at EAE day 14 captured peak disease in WT mice, while representing a potentially earlier disease phase (onset period) in *Mmp9^-/-^* mice. Only WT and *Mmp9*^-/-^ mice with similar disease scores were analysed by snRNA-seq. We obtained single-nuclei transcriptomes (henceforth referred to as cells) of dural cells from EAE WT and EAE *Mmp9^-^*^/-^ mice and integrated a naïve WT murine dural dataset as control. Cell clusters transcriptionally identified as pinealocytes (34), contaminating astrocytes (35), choroid plexus epithelial cells (36), and low-quality cells were excluded (**Supplementary Table 1**) (see Methods). We thereby retained and analytically integrated a total of 20,804 high quality single-nuclei transcriptomes from naïve WT (n = 3,096 nuclei), EAE WT (n = 4,208 nuclei) and EAE *Mmp9^-^*^/-^ (n = 8,236 nuclei) conditions.

**Figure 2:**
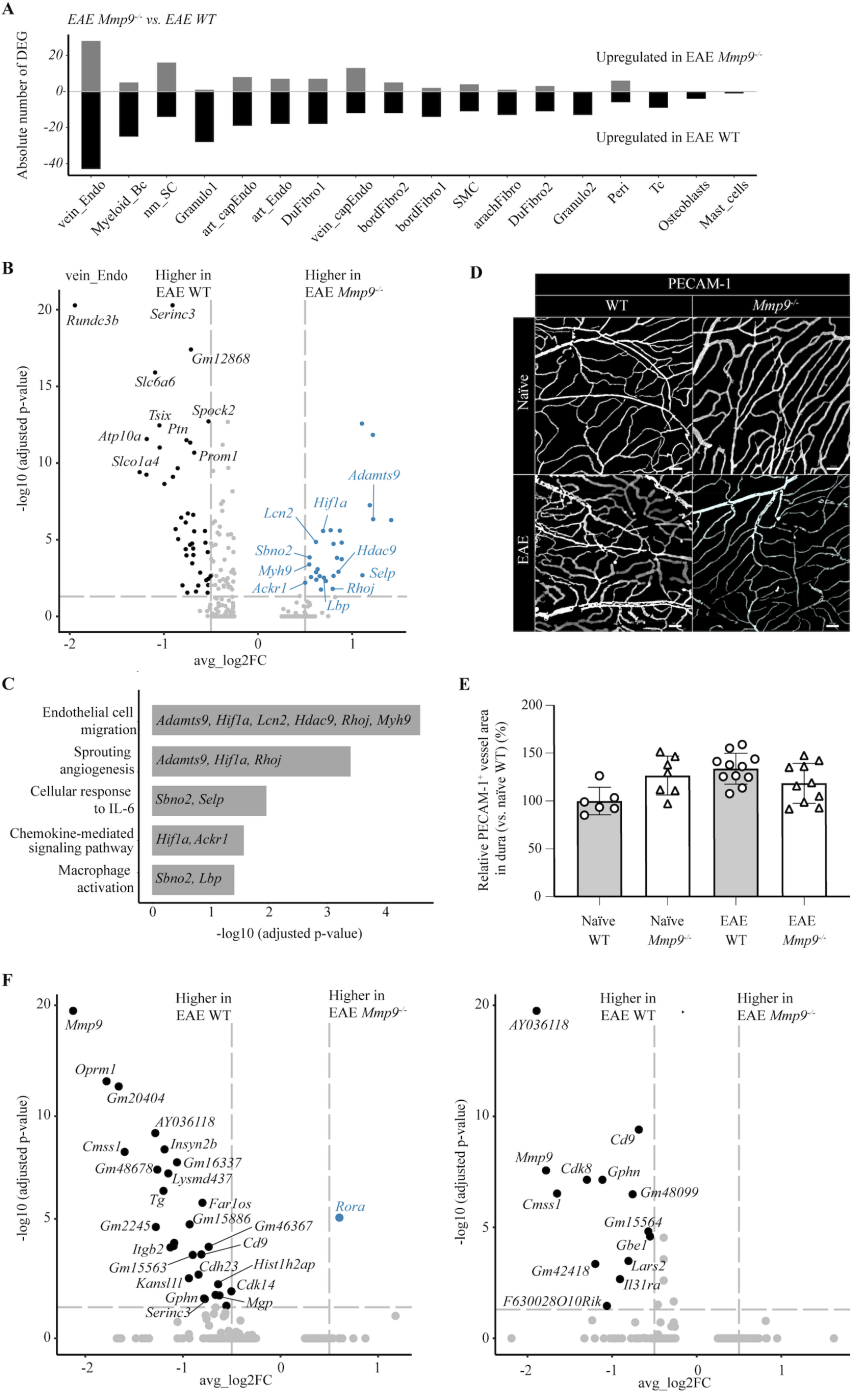
Identification of *Mmp9* expression in naïve and EAE murine dura. **(A)** Merged Uniform Manifold Approximation and Projection (UMAP) plot displaying 21 cell clusters of the combined dataset from dura of naïve WT, EAE WT and EAE *Mmp9*^-/-^ mice. **(B)** A feature plot depicting the expression of *Mmp9* in the combined dataset. **(C)** Feature plots depicting the expression of *Mmp9* in the respective conditions. **(D)** A directional bar plot illustrating the (log_2_) fold changes in relative abundance of distinct immune cell clusters between EAE *Mmp9*^-/-^ vs. EAE WT comparisons. **(E)** Volcano plot illustrating the differentially expressed genes between Granulo_1 and Granulo_2 (Granulo_2a and 2b); Log_2_ fold change >0.5 and adjusted *p* value <0.05 considered to be differentially expressed. **(F)** Selected enriched GO terms in Granulo_1 vs. Granulo_2.

After unsupervised clustering using all the cells, we identified 21 transcriptionally distinct cell clusters that were present across all conditions (**Figure 2A**, **Supplementary Figure 2A**). Among non-immune populations, two clusters corresponded to dural fibroblasts (DuFibro_1: *Col1a1*, *Runx2*; DuFibro_2: *Scara5*, *Epha3*), and two border fibroblasts (BordFibro_1 and BordFibro_2; *Slc4a10*, *Slc47a1*), as well as a cluster of arachnoid fibroblasts (arachFibro; *Alcam*, *Aldh1a2*, *Ptgds*) sharing transcriptional signatures with previously described meningeal fibroblast-like cells (12) (**Supplementary Figure 2B**). Within BordFibro_1, we detected a subpopulation expressing arachnoid barrier cell markers (*Cdh1*, *Cldn11*, *Klf5*, *Dpp4*), consistent with an arachnoid barrier phenotype (12, 37). One cluster transcriptionally resembled osteoblasts (*Ibsp*, *Bglap*, *Sparc*) (12). Endothelial cell populations were represented by five clusters expressing Pecam1 (**Supplementary Figure 2B**). One cell cluster with *Flt4* and *Prox1* expression was annotated as lymphatic endothelial cell (LymEndo) (11), while the remaining clusters were assigned to vascular endothelial phenotypes along an arterial-to-venous transcriptional continuum: arterial (art_Endo; *Arl15*, *Cdh13*, *Efnb2*), arterial-capillary (art_capEndo; *Flt1*, *Hmcn1*, *Insr*), venous-capillary (vein_capEndo), and venous (vein_Endo; *Il1r1*, *Lcn2*, *Pla2g4a*) (**Supplementary Figure 2C, D**), as described previously (38). Notably, *Slco2a1*, and *F8* were enriched in vein_capEndo, consistent with dural sinus endothelial identity, whereas vein_Endo expressed leptomeningeal endothelial cell markers (*Tjp1*, *Bsg*, *Slc2a1*) (39) (**Supplementary Figure 2E**).

Additional non-immune populations included pericytes (Peri; *Rgs5*, *Pdgfrb*), smooth muscle cells (SMC; *Myh11*, *Acta2*), and myelinating (m_SC; *Mpz*) and non-myelinating Schwann cells (nm_SC; *Cdh19*). Immune cell clusters comprised myeloid cells (Myeloid; *Lyz2*, *Mrc1*), T cells (Tc; *Cd3e*, *Il7r*), two granulocyte subsets (Granulo1 and Granulo2; *Hp*, *S100a9*), mast cells (Mast_cells; *Cpa3*), and osteoclasts (Osteoclasts; *Ctsk*). This dataset, thus, reconstructs the expected cellular landscape of the murine dura under naïve and inflammatory conditions (**Supplementary Figure 2B**).

### Identifying cellular sources of MMP-9 in murine dura

We next characterized the cellular sources of gelatinase gene expression. As expected, *Mmp9* transcripts were detected in the combined dataset, while no expression was observed in *Mmp9^-^*^/-^ transcriptomes, confirming effective genetic ablation (**Figures 2B, C**; **Supplementary Figure 2F**). In non-diseased dura snRNA-seq data, *Mmp9* expression was primarily restricted to immune cell clusters, including Granulo1, Granulo2, and osteoclasts (**Figures 2B, C**). This expression pattern is consistent with previous findings in other tissues (9, 18, 40, 41), indicating that *Mmp9* is expressed by specific myeloid cell populations under homeostatic conditions.

### Mmp9 deficiency reshapes meningeal cell composition

Gelatinases facilitate lymphocyte penetration of the parenchymal border of the BBB and play various roles, including activation or inactivation of chemokine gradients and selective cleavage of cell-cell junction molecules and receptors for BM components (8). However, the functional relevance of gelatinases in the meninges is poorly understood. We therefore assessed whether *Mmp9* affects the composition of meningeal cell populations during EAE. Comparative analysis of *Mmp9^-/-^* and WT EAE mice revealed the most pronounced alterations in the composition of dural cell populations. Clusters associated with barrier localization and function, including bordFibro1 and arachFibro, were relatively enriched in *Mmp9^-/-^* EAE samples, whereas clusters with predicted dural fibroblast identity were reduced (**Supplementary Figure 2A**). This was accompanied by an increased abundance of endothelial cell clusters annotated as venous and venous capillary endothelial cells, along with a reduced representation of T cell populations (**Figure 2D**, **Supplementary Figure 2A**). Notably, changes were also detected in cells that do not express *Mmp9*, consistent with the secreted nature of MMPs and their varied effects on the microenvironment. Fold differences (log_2_) revealed the most prominent changes between EAE *Mmp9^-/-^* and EAE WT samples (**Figure 2D**) within the granulocyte compartment, characterized by an increase in the Granulo1 cluster and a concomitant reduction in the Granulo2 cluster (**Figure 2D**). Comparisons with WT naïve confirmed that the Granulo1 and Granulo2 undergo the most substantial alterations upon EAE induction (**Supplementary Figure 3**). This shift likely reflects a phenotypic reprogramming of granulocytes in the absence of MMP-9 during neuroinflammation. To better understand the identity and functional differences between these two granulocyte populations, we performed differential gene expression analysis followed by GO term enrichment (**Figures 2E, F**). While both clusters share a myeloid signature, Granulo1 cells were characterized by the upregulation of genes associated with regulatory and homeostatic processes, such as *Prkg1*, *Ptprj*, and *Foxp1*. By contrast, Granulo2 cells, which were enriched in *Mmp9^-/-^* EAE dura, showed a transcriptional profile consistent with an activated, pro-inflammatory neutrophil phenotype rather than a distinct cell type. GO terms enriched in this cluster included defense response to bacterium, cell chemotaxis, reactive oxygen species (ROS), metabolic processes, antimicrobial humoral responses, and phagocytosis. These pathways were driven by upregulated expression of classical neutrophil activation markers such as *Il1b*, *S100a8*, *S100a9*, *Lcn2*, *Cyba*, and *Ncf4*, among others (**Figures 2E, F**). These results suggest that *Mmp9* deficiency in EAE promotes a functional shift in dura-associated granulocytes toward a more inflammatory phenotype rather than the appearance of a different cell type.

### Angiogenic and migratory phenotype induced in dura venous endothelial cells by Mmp9 deficiency

**Figure 3:**
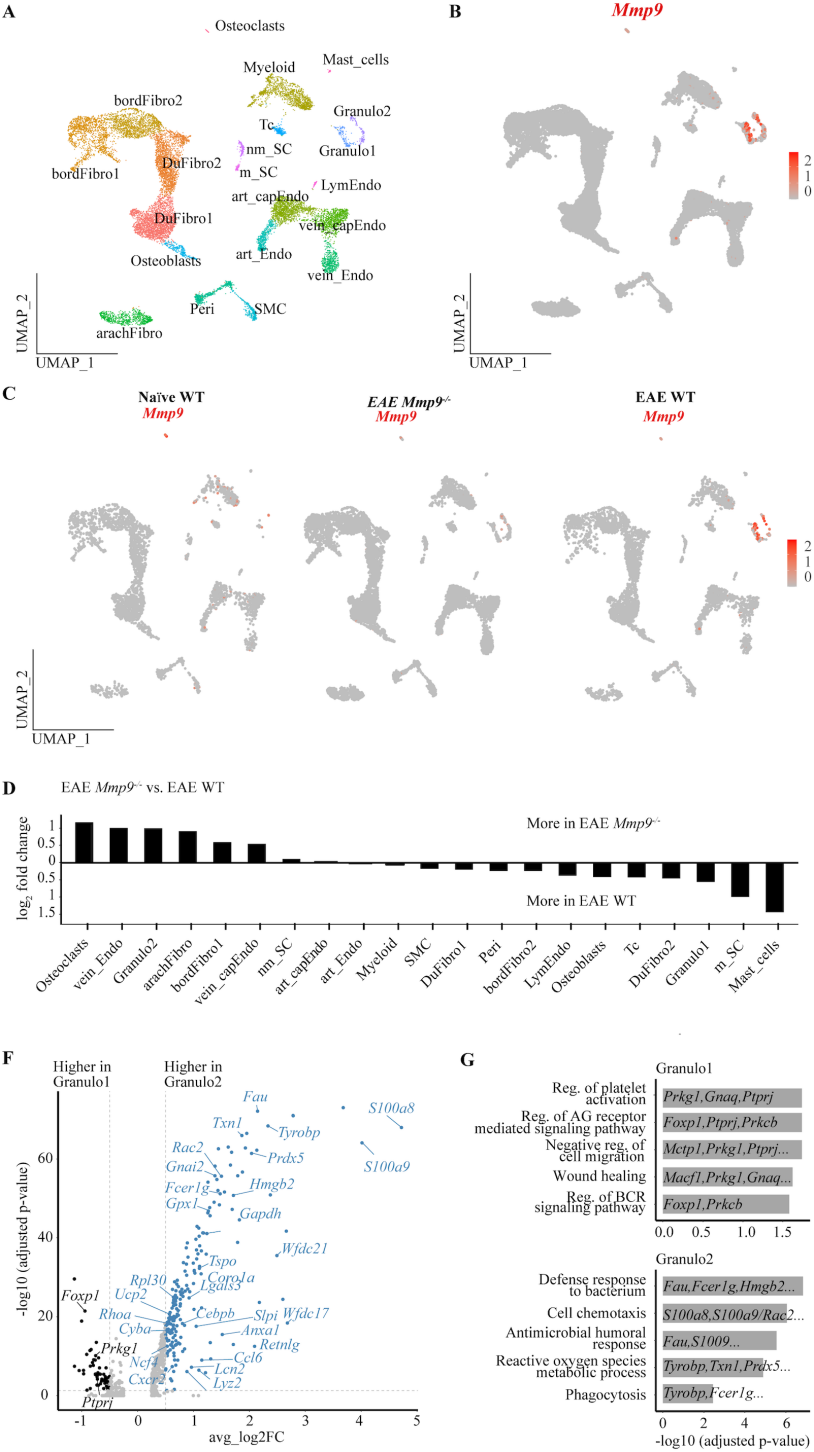
*Mmp9* deficiency triggered transcriptional changes in murine dura during EAE. **(A)** Stacked bar plots showing the number of differentially expressed genes in individual cell clusters in EAE *Mmp9*^-/-^ vs. EAE WT comparison. **(B)** Volcano plots depicting differentially expressed genes in vein_Endo in EAE *Mmp9*^-/-^ vs. EAE WT. **(C)** Selected GO terms mediated by upregulated DEGs in EAE *Mmp9*^-/-^ vs. EAE WT. **(D)** Representative images of whole-mount dura immunofluorescently stained for PECAM-1, showing blood vessels. Scale bars = 100 µm. **(E)** PECAM-1^+^ vessel area in dura was analyzed using Imaris and normalized to naïve WT. Each dot represents an individual mouse. N = 6 – 11; data are presented as means ± SD. **(F)** Volcano plots depicting differentially expressed genes for Granulo1 in EAE *Mmp9*^-/-^ vs. EAE WT. **(G)** Volcano plots depicting differentially expressed genes for Granulo2 in EAE *Mmp9*^-/-^ vs. EAE WT.

We next tested how *Mmp9* deficiency affected the transcriptome (as opposed to the relative abundance) of individual cell clusters in EAE. In the comparison between EAE *Mmp9^-/-^* and EAE WT mice, differentially expressed genes were detected across most cell clusters, with the highest number observed in the vein_Endo cluster (Up:28; Down:43) (**Figures 3A, B**). GO term enrichment analysis identified that upregulated genes in EAE *Mmp9^-/-^* mice, including *Adamts9, Hif1a, Lcn2, Hdac9, Rhoj*, and *Myh9*, were associated with endothelial cell migration. Additionally, *Adamts9*, *Hif1a*, *Hdac9*, *Rhoj*, and *Tcf4* were linked to angiogenesis (**Figure 3C**). Venous endothelial cells were, thus, among the most responsive cell types to *Mmp9* deficiency during EAE. However, immunofluorescence staining for PECAM-1 in the dura of *Mmp9^-/-^* vs. WT mice did not show significant changes in vessel density in either naïve or EAE conditions (**Figures 3D, E**), suggesting that potential vascular changes are subtle or confined to specific vessel subsets.

Analysis of the Granulo1 and Granulo2 populations in WT EAE versus *Mmp9^-/-^* EAE revealed that most differentially expressed genes were downregulated in the *Mmp9^-/-^* Granulo1 and Granulo2 populations (**Figures 3F, G**). The only gene upregulated under *Mmp9^-/-^* EAE conditions was *Rora* in the Granulo1 cluster, a transcription factor normally associated with T lymphocytes but which has implicated in diverse biological processes (42). However, GO term enrichment analysis did not identify any significantly enriched biological pathways in either Granulo1 or Granulo2 in either WT and *Mmp9^-/-^* under EAE conditions.

### Mature granulocytes accumulate in the dura of Mmp9^-/-^ mice during EAE

**Figure 4:**
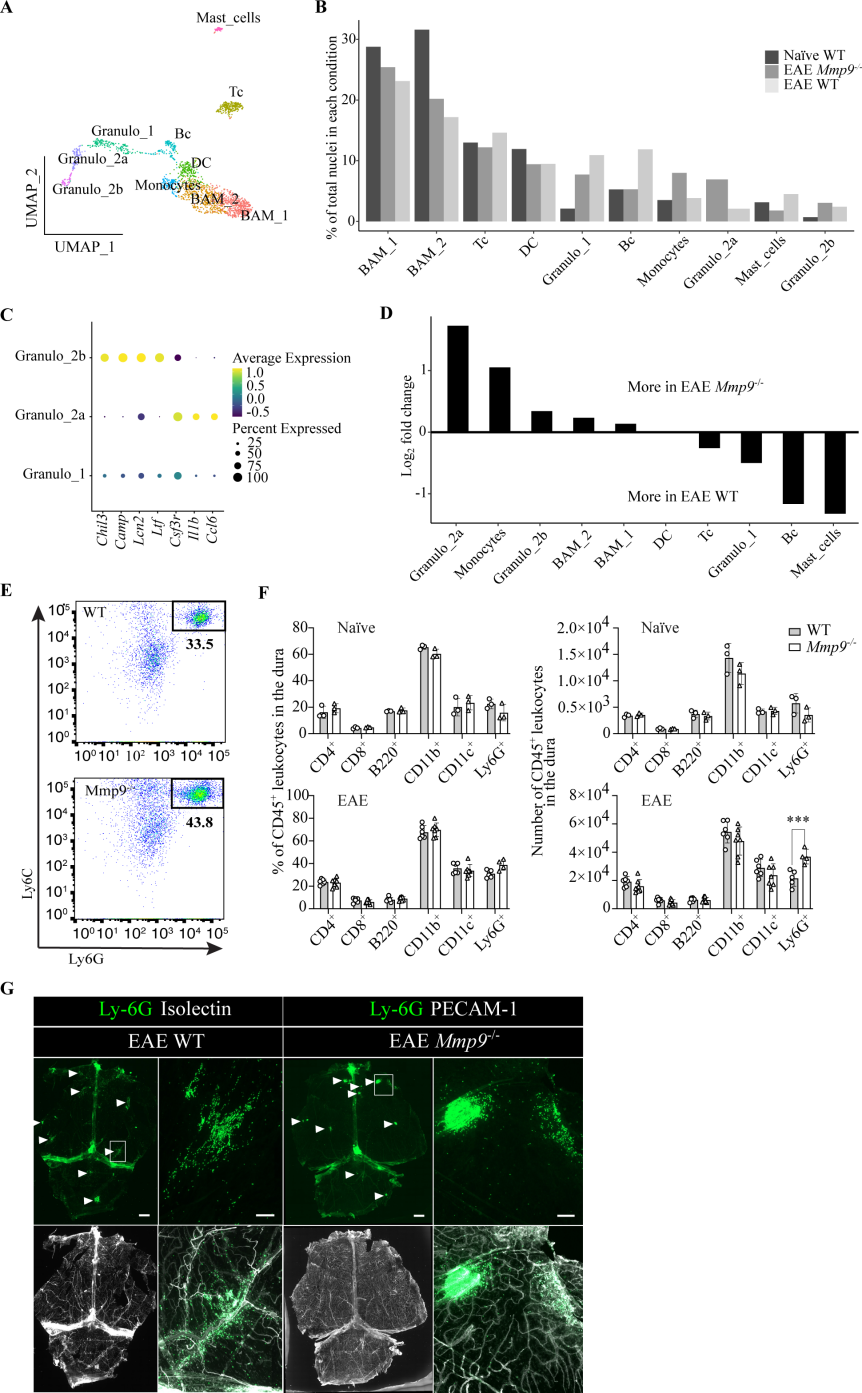
Immune cell responses in murine dura to gelatinase deficiencies during EAE. **(A)** UMAP plot of immune cell clusters from dura of naïve and EAE WT (naïve WT, n = 285 nuclei; EAE WT, n = 623 nuclei) and EAE *Mmp9* deficient (EAE *Mmp9*^-/-^, n = 1114 nuclei) mice. **(B)** Bar plot depicting the relative abundance of individual immune cell clusters in the respective conditions. **(C)** Dot plot of maturity related genes in granulocyte populations. Dot size depicts percentage of cells within a cell cluster expressing the selected genes, and color defines the average expression level of the selected genes. **(D)** A directional bar plot illustrating the (log_2_) fold changes in relative abundance of distinct immune cell clusters between EAE *Mmp9*^-/-^ vs. EAE WT comparisons. Bars oriented upwards indicating increased relative abundance in the dura of EAE *Mmp9*^-/-^ mice, whereas the downwards-oriented bars indicating increased relative abundance in the dura of EAE WT mice. **(E)** Representative gating strategy for identification of Ly6C^+^Ly6G^+^ neutrophils in the dura by flow cytometry. **(F)** Quantification of leukocyte subtypes in the dura of naïve and EAE induced WT and *Mmp9*^-/-^ mice. Each data point represents a pool of three dura samples. Statistical analysis was performed using two-way ANOVA, ***p < 0.001. **(G)** Representative images of whole-mount dura immunofluorescence staining for Ly-6G to mark neutrophils and PECAM-1 or isolectin B4 to mark blood vessels. Scale bars = 1 mm (in lower magnifications) and 200 µm (in higher magnifications).

We next performed higher-resolution sub-clustering to characterize immune cell responses to EAE in the absence of MMP-9. The 2,736 *Ptprc*^+^ nuclei from the combined dataset segregated into distinct leukocyte subclusters based on known marker genes (**Figure 4A**, **Supplementary Figure 4A**). Lymphoid lineage clusters comprised T cells (*Gata3*, *Cd3e*, *Cd3g*) and B cells (*Cd79a*, *Ms4a1*). Among myeloid lineage clusters (43), two populations were characterized by macrophage-associated transcripts and corresponded to BAM_1 and BAM_2 (*Lyz2*). Both clusters exhibited a border-associated phenotype (*Mrc1*, *Pf4*, *Lyve1*) (18). BAM_2 (*H2-Eb1/H2-Aa/H2-Ab1*, *Cd74*, *Ctss*) displayed a pronounced MHC-II–mediated antigen-presentation program, whereas BAM_1 (*F13a1*, *Slc9a9*, *Mctp1*) exhibited a transcriptional profile linked to tissue homeostasis, including extracellular matrix stabilization and endosomal trafficking, consistent with a homeostatic, repair-oriented macrophage state (**Supplementary Figure 4B**). Additional clusters expressed monocyte-associated markers (*Vcan, Ccr2*) and three distinct granulocyte clusters were identified (Granulo_1, Granulo_2a, Granulo_2b; *Lyz, Hp, S100a9*). Granulo_2a cells were absent in naïve WT dura (**Figure 4B**) and displayed higher expression of *Csf3r*, *Il1b*, and *Ccl6*, indicative of mature neutrophils (44), whereas Granulo_2b displayed higher expression of *Chil3*, *Camp*, *Lcn2* and *Ltf* indicative of bone marrow–associated neutrophils (44) (**Figure 4C**). GO term analysis of marker genes suggested that Granulo_2a exhibit immunoregulatory features (**Supplementary Figure 4C**). In *Mmp9^-^*^/-^ vs. WT EAE, the largest relative increase was observed in Granulo_2a (+4.83%), followed by monocytes (+4.14%), BAM_2 (+3.02%), BAM_1 (+2.29%), and Granulo_2b (+0.64%). By contrast, B cells, Granulo_1, mast cells, and T cells were reduced in relative abundance compared to WT (**Figure 4D**). The most prominent shift induced by *Mmp9* deficiency was a redistribution from Granulo_1 to Granulo_2 populations. Additional subclusters corresponded to dendritic cells (*Cd209a, Clec9a*) and mast cells (*Tpsb2, Cpa3*).

To validate these transcriptomic findings, we performed flow cytometry of immune cell populations in the dura of naïve and EAE-induced *Mmp9^-/-^* and WT mice (**Figures 4E, F**). No differences in immune cell composition were detected in naïve animals, indicating the absence of baseline alterations. Consistent with the snRNA-seq results, Ly6C^+^Ly6G^+^ neutrophils were more abundant in the dura of *Mmp9^-/-^* EAE mice compared to WT EAE controls (**Figure 4F**). Immunofluorescence staining for Ly6G together with isolectin and PECAM-1 revealed focal Ly6G^+^ cell accumulations in the dura of both genotypes; these foci appeared more frequent and larger in *Mmp9^-/-^* mice (**Figure 4G**). Collectively, these data indicate that *Mmp9* deficiency during EAE promotes expansion of myeloid cell populations, particularly mature neutrophils, and alters the composition of granulocytic infiltrates within the dura.

## Discussion

In this study, we identify MMP-9 as a selective and dynamically regulated gelatinase in the murine dura during neuroinflammation. We show that both MMP-2 and MMP-9 are present under homeostatic conditions, but only MMP-9 is markedly upregulated during EAE. *Mmp9^-/-^* mice exhibited a delayed onset of clinical symptoms, but developed more severe disease at peak, a phenotype accompanied by pronounced alterations in dural immune cell composition and endothelial transcriptional states. Single-nucleus RNA-seq revealed that *Mmp9* deficiency led to a redistribution of granulocyte subsets toward a mature, pro-inflammatory neutrophil phenotype, as well as transcriptional activation of venous endothelial cells toward migratory and angiogenic programs. Flow cytometry and immunohistology confirmed increased neutrophil accumulation in the dura of *Mmp9^-/-^* mice. These findings suggest that, in the dura, MMP-9 may exert context-dependent, potentially anti-inflammatory functions, which contrast with its more barrier-modulating pro-inflammatory role at the BBB (9, 33).

Venous endothelial cells were among the most transcriptionally responsive cell types to *Mmp9* deficiency in EAE, displaying upregulation of genes associated with migration (*Adamts9, Hif1a, Hdac9, Rhoj, Myh9*) and angiogenesis (*Adamts9, Hif1a, Tcf4*). Such signatures are consistent with previous reports implicating MMP-9 in vascular remodeling and angiogenic processes through ECM remodeling and growth factor release (2, 6). However, despite these transcriptional changes, we did not observe overt differences in vessel density between genotypes, suggesting that endothelial adaptations may be subtle, temporally restricted, or confined to specific vascular segments. The altered endothelial state may also reflect secondary responses to changes in immune cell composition or cytokine *milieu*, as *Mmp9* deficiency has been linked to altered systemic cytokine and chemokine levels during EAE (45).Thus, vascular phenotypes in *Mmp9^-/-^* dura may arise from a combination of direct MMP-9-dependent effects and indirect effects mediated by the inflammatory microenvironment.

The most striking immune alteration in *Mmp9^-/-^* dura was a shift from Granulo1 to Granulo2 populations, with Granulo_2a cells exhibiting a mature neutrophil transcriptome (*Csf3r, Il1b, Ccl6*) and enriched pro-inflammatory pathways, including defense responses to bacteria, ROS metabolism, and chemotaxis. These changes were paralleled by a significant increase in Ly6C^+^Ly6G^+^ neutrophils by flow cytometry. Such findings align with earlier work showing that MMP-9 modulates neutrophil recruitment and activation, in part through proteolytic processing of chemokines such as CXCL8/IL-8, CXCL1, and CXCL2, thereby shaping chemokine gradients (1, 46, 47). MMP-9-mediated chemokine cleavage can have both activating and inactivating effects, depending on context, suggesting that its absence could prolong or intensify neutrophil-attracting signals in the dura (9, 48).

The significance of these changes may extend beyond local effects. MMP-9-expressing granulocytes are abundant in the periphery, and systemic alterations in cytokine and chemokine levels have been reported in *Mmp9^-/-^* mice during EAE (45). This, together with the primary granulocytic source of MMP-9 in our dataset, suggests that peripherally derived MMP-9 contributes to establishing an effective and temporally regulated immune response. In support of this possibility, WT mice reconstituted with MMP-2/MMP-9 double knockout bone marrow display predominantly peripheral immune defects (9, 10). In the dura, absence of MMP-9 may thus impair resolution of neutrophil recruitment signals, leading to sustained accumulation of mature neutrophils.

The dual-phase EAE phenotype observed in *Mmp9^-/-^* mice, delayed onset but more severe peak, may be directly related to the temporal and qualitative changes in granulocyte recruitment we observe. Early in disease, the absence of MMP-9 could delay immune cell penetration or alter chemokine gradient formation, slowing initial disease progression. However, as inflammation advances, unmodulated chemotactic cues and endothelial activation in the dura may favor excessive neutrophil accumulation, amplifying local and possibly systemic inflammation, thereby exacerbating disease severity at peak. This model reconciles the seemingly paradoxical early protective and later deleterious effects of *Mmp9* deficiency, highlighting a context-dependent role for MMP-9 in neuroinflammation that differs between CNS border compartments.

Beyond MMP-2 and MMP-9, other MMP family members also contribute to CNS barrier regulation and inflammation. Notably, MMP-14 (MT1-MMP) has recently been shown to mediate cerebrospinal fluid barrier dysfunction and immune cell trafficking (49). Together, these findings highlight the broader roles of MMPs in brain pathophysiology, including inflammation, vascular remodeling, and tumor progression, and emphasize the need for context-specific understanding when targeting MMPs therapeutically.

Our findings underscore the importance of the dura as a distinct immunological niche during CNS inflammation and reveal that MMP-9, beyond its established role at the BBB, modulates the composition and activation state of dural immune and endothelial populations. The apparent anti-inflammatory function of MMP-9 in the dura raises caution for therapeutic strategies aiming to broadly inhibit MMP-9 in neuroinflammatory conditions. Future work should dissect the temporal dynamics of MMP-9 activity in distinct CNS border tissues and clarify its mechanistic interplay with chemokine networks and endothelial signaling, ideally integrating longitudinal single-cell and functional approaches in both murine and human systems.

## Data Availability

The data presented in the study are deposited in the European Nucleotide Archive (ENA), accession numbers PRJEB101900 and PRJEB102008.
