# Supplementary material for: MMP-9 affects dural cell composition and granulocyte accumulation during neuroinflammation
Source: Front Immunol. 2025 Dec 11;16:1713166. doi: 10.3389/fimmu.2025.1713166 (PMC12738929; doi:10.3389/fimmu.2025.1713166)
Supplement: Supplementary file 1 [file DataSheet1.pdf]

Supplementary Material

Supplementary Figures and Tables

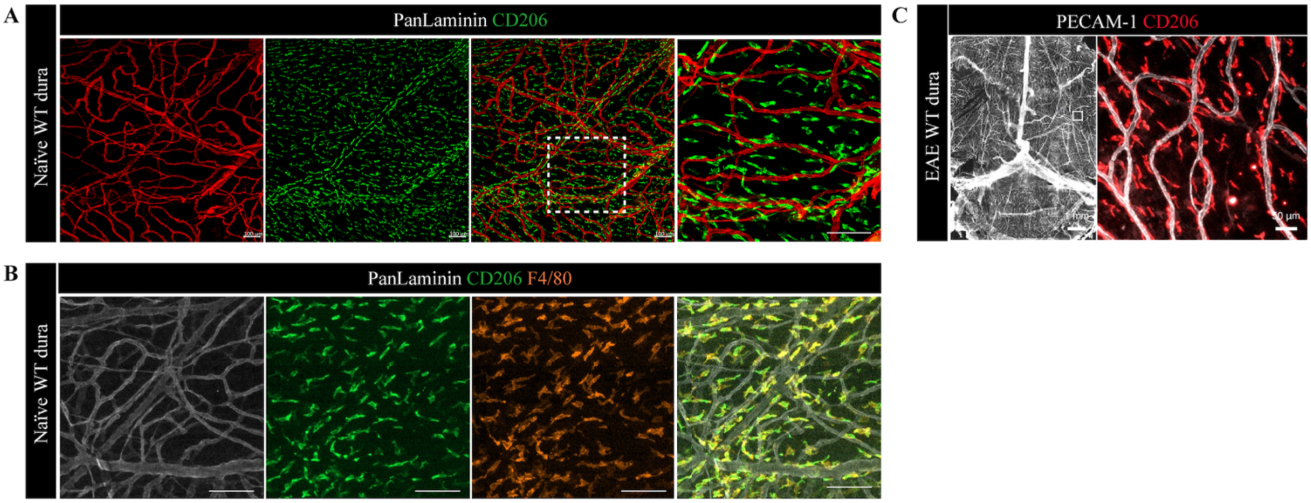

Supplementary Figure 1: Identification of total and resident macrophage populations in the dura mater under naïve and EAE conditions.

(A) Whole-mount immunofluorescence staining of dura mater from naïve WT mice with pan laminin antibody (Pan LM) to mark basement membranes (red) and anti-CD206 to mark resident macrophages (green). Boxed area is shown at higher magnification to the right. Scale bars = 100 µm.
(B) Whole-mount immunofluorescence staining of dura mater from naïve WT mice with pan laminin antibody (Pan LM) (white) and anti-F4/80 as a general macrophage marker (orange). Scale bars = 100 µm.
(C) Whole-mount immunofluorescence staining of dura mater from peak EAE WT mice with anti-PECAM-1 antibody to mark blood vessels (white) and anti-CD206 (red). Boxed area is shown at higher magnification to the right. Scale bars are indicated.

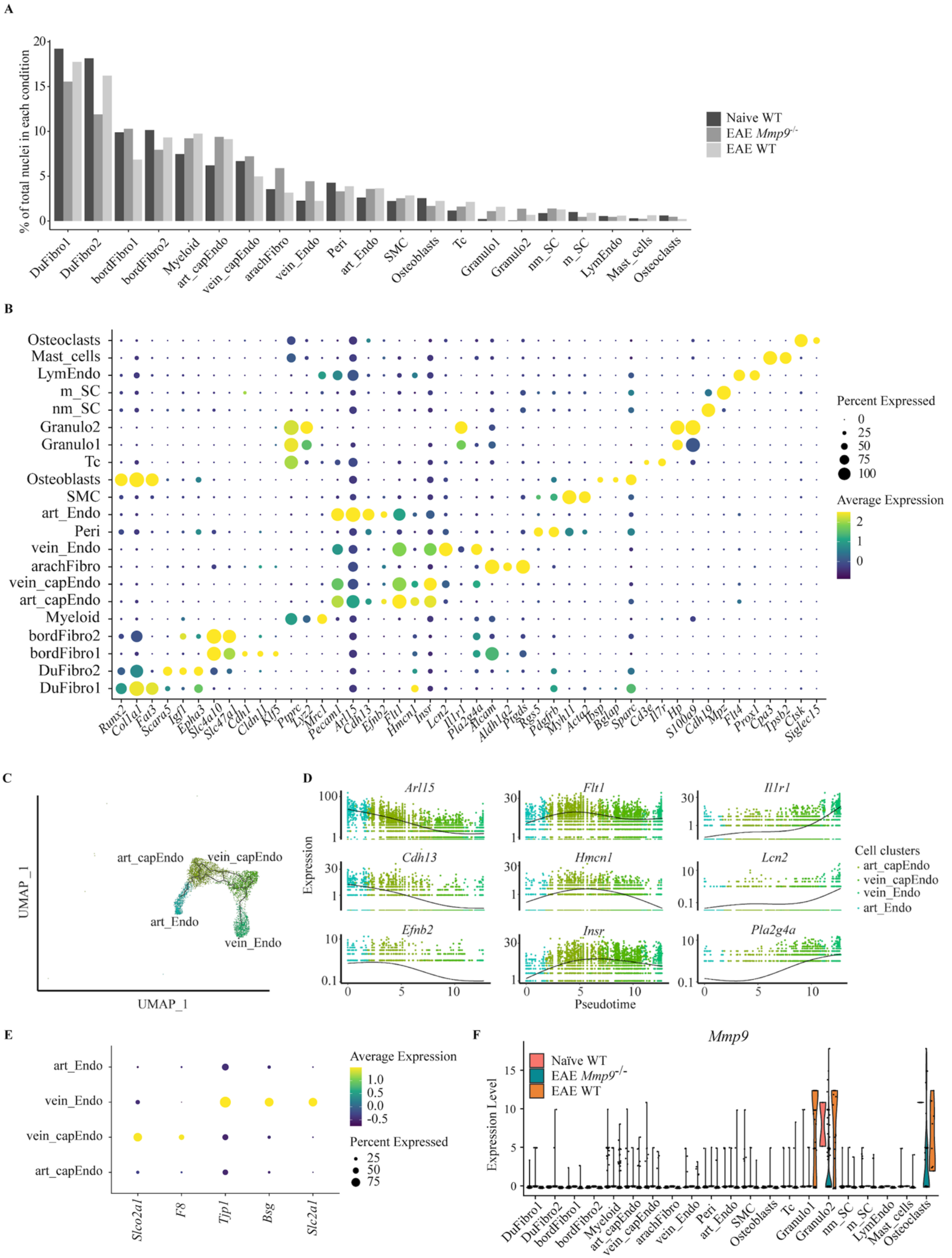
A
% of total nuclei in each condition
20
15
10
5
0
Naive WT
EAE Mmp9-/-
EAE WT
DuFibro1
DuFibro2
bordFibro1
bordFibro2
Myeloid
art_capEndo
vein_capEndo
arachFibro
vein_Endo
Peri
art_Endo
SMC
Osteoblasts
Tc
Granulo1
Granulo2
nm_SC
m_SC
LymEndo
Mast_cells
Osteoclasts
B
Osteoclasts
Mast_cells
LymEndo
m_SC
nm_SC
Granulo2
Granulo1
Tc
Osteoblasts
SMC
art_Endo
Peri
vein_Endo
arachFibro
vein_capEndo
art_capEndo
Myeloid
bordFibro2
bordFibro1
DuFibro2
DuFibro1
Runx2
Col1a1
Fat3
Scara5
Igf1
Epha3
Slc4a10
Slc47a1
Cdh1
Cldn11
Klf5
Ptprc
Lyz2
Mrc1
Pecam1
Arl15
Cdh13
Efnb2
Flt1
Hmcn1
Insr
Lcn2
Il1r1
Pla2g4a
Alcam
Aldh1a2
Ptgds
Rgs5
Pdgfrb
Myh11
Acta2
Ibsp
Bglap
Sparc
Cd3e
Il7r
Hp
S100a9
Cdh19
Mpz
Flt4
Prox1
Cpa3
Tpsb2
Ctsk
Siglec15
Percent Expressed
0
25
50
75
100
Average Expression
2
1
0
C
UMAP_1
art_capEndo
vein_capEndo
art_Endo
vein_Endo
UMAP_1
D
Expression
Arl15
Flt1
Il1r1
Cdh13
Hmcn1
Lcn2
Efnb2
Insr
Pla2g4a
Pseudotime
Cell clusters
art_capEndo
vein_capEndo
vein_Endo
art_Endo
E
art_Endo
vein_Endo
vein_capEndo
art_capEndo
Slco2a1
F8
Tjp1
Bsg
Slc2a1
Average Expression
1.0
0.5
0.0
-0.5
Percent Expressed
25
50
75
F
Mmp9
Naïve WT
EAE Mmp9-/-
EAE WT
Expression Level
15
10
5
0
DuFibro1
DuFibro2
bordFibro1
bordFibro2
Myeloid
art_capEndo
vein_capEndo
arachFibro
vein_Endo
Peri
art_Endo
SMC
Osteoblasts
Tc
Granulo1
Granulo2
nm_SC
m_SC
LymEndo
Mast_cells
Osteoclasts

Supplementary Figure 2: Transcriptional characterization of dural resident cells.

(A) Bar plot depicting the relative abundance of individual cell clusters in the respective conditions.
(B) Dot plot of marker genes defining the all identified cell clusters in naïve WT, and EAE WT and *Mmp9*$^{-/-}$ dura. Dot size depicts percentage of cells within a cell cluster expressing the selected genes, and color defines the average expression level of the selected gene.
(C) Pseudotime analysis of vascular endothelial cell clusters, including cells transcriptionally resembling arterial and capillary endothelial cells (art_capEndo), venous and capillary (vein_capEndo), venous endothelial cells (vein_Endo), and arterial endothelial cells (art_Endo).
(D) Expression of selected marker genes for arterial (*Arl15*, *Cdh13*, *Efnb2*), capillary (*Flt1, Hmcn1, Insr*) and venous (*Illr1, Lcn2, Pla2g4a*) endothelial cells along the inferred trajectory corresponding to (C).
(E) Expression patterns of *Slco2a1* and *F8* in vein_capEndo consistent with dural sinus endothelial identity, and *Tjp1*, *Bsg*, *Slc2a1* enrichment in vein_Endo consistent with leptomeningeal endothelial phenotype.
(F) Violin plots of *Mmp9* expression levels in the individual cell clusters in naïve WT, and EAE WT and *Mmp9*$^{-/-}$ dura.

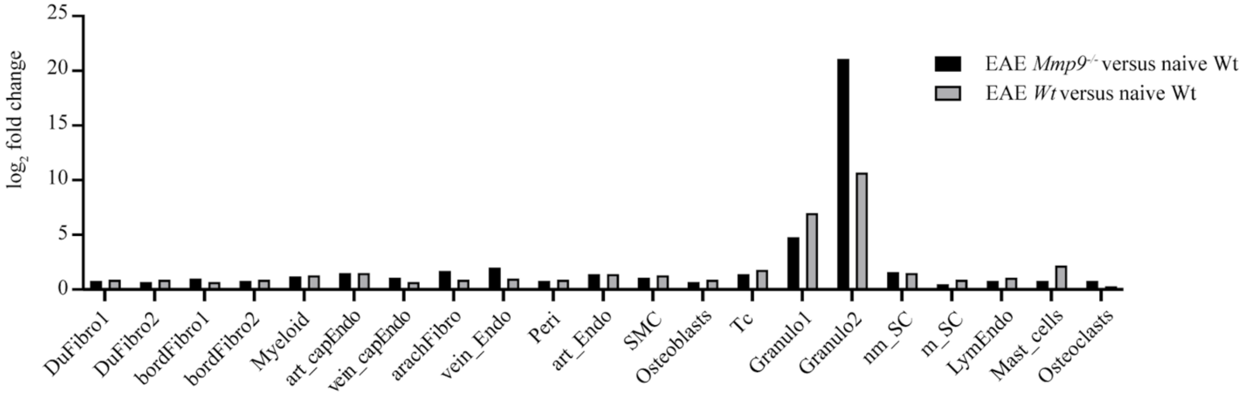

Supplementary Figure 3: Selective increase of granulocytes in EAE Dura.

A bar plot illustrating the (log_2) fold changes in relative abundance of distinct cell clusters comparing naïve WT with EAE WT or EAE *Mmp9*$^{-/-}$.

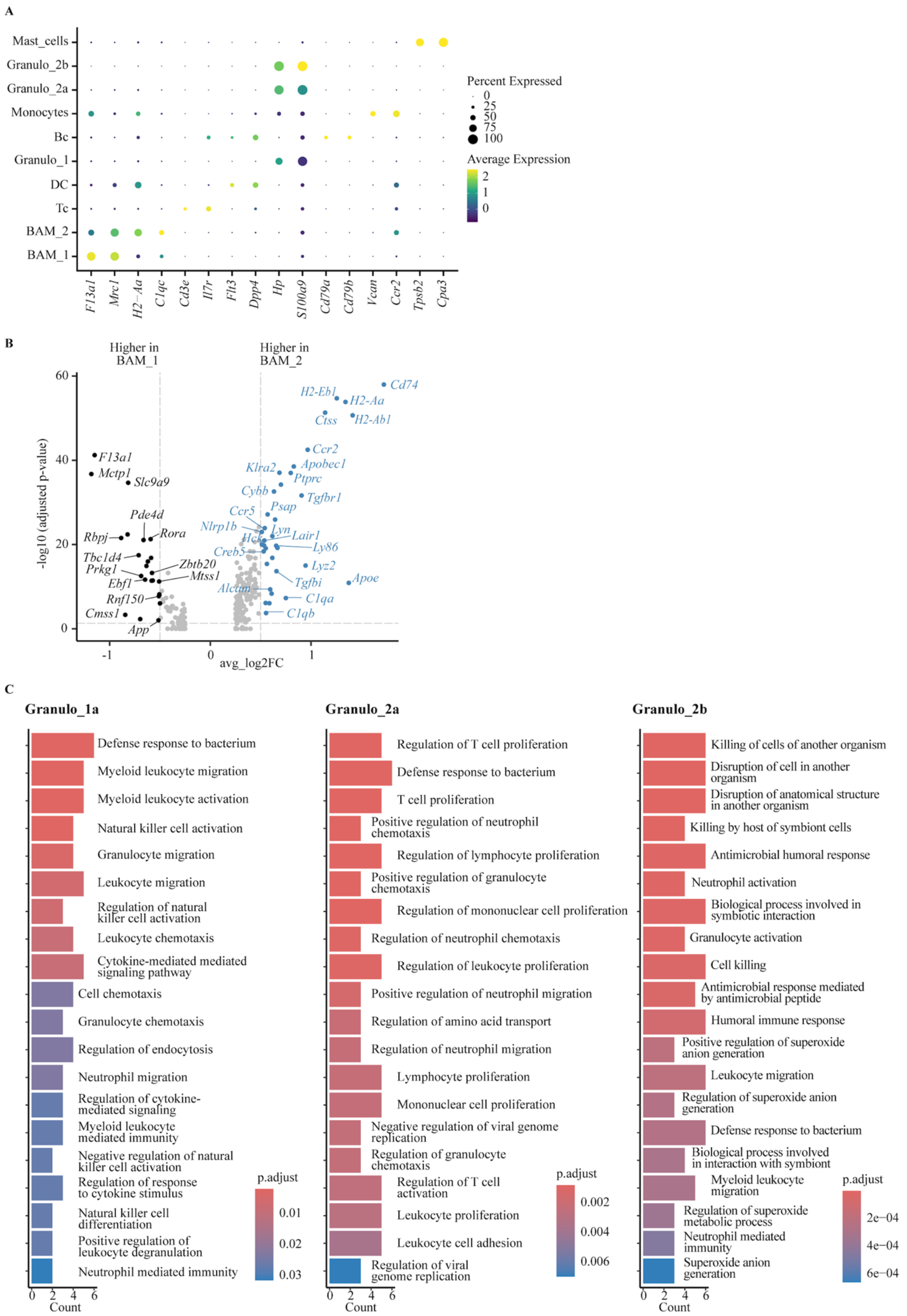

A
Mast_cells
Granulo_2b
Granulo_2a
Monocytes
Bc
Granulo_1
DC
Tc
BAM_2
BAM_1
F13a1
Mrc1
H2-Aa
C1qc
Cd3e
Il7r
Flt3
Dpp4
Hp
S100a9
Cd79a
Cd79b
Vcan
Ccr2
Tpsb2
Cpa3
Percent Expressed
0
25
50
75
100
Average Expression
2
1
0
B
Higher in BAM_1
Higher in BAM_2
-log10 (adjusted p-value)
avg_log2FC
60
40
20
0
-1
0
1
Cd74
H2-Eb1
H2-Aa
Ctss
H2-Ab1
Ccr2
Apobec1
Klra2
Ptprc
Cybb
Tgfbr1
Psap
Ccr5
Nlrp1b
Lyn
Lair1
Hck
Ly86
Creb5
Lyz2
Tgfbi
Apoe
Alcam
C1qa
C1qb
F13a1
Mctp1
Slc9a9
Pde4d
Rbpj
Rora
Tbc1d4
Zbtb20
Prkg1
Mtss1
Ebf1
Rnf150
Cmss1
App
C
Granulo_1a
Defense response to bacterium
Myeloid leukocyte migration
Myeloid leukocyte activation
Natural killer cell activation
Granulocyte migration
Leukocyte migration
Regulation of natural killer cell activation
Leukocyte chemotaxis
Cytokine-mediated mediated signaling pathway
Cell chemotaxis
Granulocyte chemotaxis
Regulation of endocytosis
Neutrophil migration
Regulation of cytokine-mediated signaling
Myeloid leukocyte mediated immunity
Negative regulation of natural killer cell activation
Regulation of response to cytokine stimulus
Natural killer cell differentiation
Positive regulation of leukocyte degranulation
Neutrophil mediated immunity
p.adjust
0.01
0.02
0.03
Count
0
2
4
6
Granulo_2a
Regulation of T cell proliferation
Defense response to bacterium
T cell proliferation
Positive regulation of neutrophil chemotaxis
Regulation of lymphocyte proliferation
Positive regulation of granulocyte chemotaxis
Regulation of mononuclear cell proliferation
Regulation of neutrophil chemotaxis
Regulation of leukocyte proliferation
Positive regulation of neutrophil migration
Regulation of amino acid transport
Regulation of neutrophil migration
Lymphocyte proliferation
Mononuclear cell proliferation
Negative regulation of viral genome replication
Regulation of granulocyte chemotaxis
Regulation of T cell activation
Leukocyte proliferation
Leukocyte cell adhesion
Regulation of viral genome replication
p.adjust
0.002
0.004
0.006
Count
0
2
4
6
Granulo_2b
Killing of cells of another organism
Disruption of cell in another organism
Disruption of anatomical structure in another organism
Killing by host of symbiont cells
Antimicrobial humoral response
Neutrophil activation
Biological process involved in symbiotic interaction
Granulocyte activation
Cell killing
Antimicrobial response mediated by antimicrobial peptide
Humoral immune response
Positive regulation of superoxide anion generation
Leukocyte migration
Regulation of superoxide anion generation
Defense response to bacterium
Biological process involved in interaction with symbiont
Myeloid leukocyte migration
Regulation of superoxide metabolic process
Neutrophil mediated immunity
Superoxide anion generation
p.adjust
2e−04
4e−04
6e−04
Count
0
2
4
6

Supplementary Figure 4: Characterization of immune cell subpopulations in murine dura. **(A)** Dot plot of marker genes for distinct immune cell subpopulation in the immune cell combined dataset.

(B) Volcano plot illustrating the differentially expressed genes between BAM_1 and BAM_2; (Log_2) Fold change > |0.5| and adjusted P value <0.05 considered to be differentially expressed. **(C)** Top 20 enriched biological processes of top differentially expressed genes. GO term analysis of top marker genes in distinct granulocytes subsets: Granulo_1, Granulo_2a, and Granulo_2b.

Supplementary Tables

Supplementary Table 1: Relative abundance of excluded cell clusters in the dura mater single-nucleus RNA-seq dataset.
The table reports the proportion of nuclei assigned to four excluded clusters - pinealocytes (PC), astrocytes (Astro), choroid plexus epithelial cells (ChP), and low-quality nuclei (LowQ) - across experimental groups. Values represent the percentage of each excluded cluster relative to the total nuclei per group. Experimental groups include control wild-type mice (Naïve *WT*) and EAE-induced mice with either *Mmp9* deficiency (EAE *Mmp9*$^{-/-}$) or wild-type background (EAE *WT*).

Cluster	Naïve *WT*	EAE *Mmp9*$^{-/-}$	EAE *WT*
PC	6.62%	6.93%	3.73%
Astro	1.31%	2.13%	1.22%
ChP	2.36%	1.84%	0.67%
LowQ	1.73%	1.06%	3.08%
